# Determination of the effects of advanced glycation end products receptor polymorphisms and its activation on structural cell responses and inflammation in asthma

**DOI:** 10.55730/1300-0144.5569

**Published:** 2022-11-30

**Authors:** Esra BİRBEN, Ümit Murat ŞAHİNER, Can Ömer KALAYCI

**Affiliations:** 1 Department of Biology, Faculty of Science, Hacettepe University, Ankara, Turkey; 2 Department of Pediatrics, Faculty of Medicine, Hacettepe University, Ankara, Turkey

**Keywords:** Asthma, airway cells, angiogenesis, RAGE, remodeling

## Abstract

**Background/aim:**

Advanced glycation end products receptor (RAGE) is a pattern recognition receptor which attracted attention in chronic airway diseases recently. This study aimed to determine the association of RAGE with asthma and the cellular responses resulting from RAGE signaling pathway activation.

**Materials and methods:**

Asthmatic (n = 362) and healthy (n = 134) children were genotyped by PCR-RFLP. Plasma sRAGE levels were determined by ELISA. Lung structural cells were stimulated with AGEs (advanced glycation end products) and control BSA. Expressions of cytokines and protein levels were determined by real-time PCR and ELISA.

**Results:**

Gly82Ser and −374 T/A polymorphisms in RAGE gene were associated with lower plasma sRAGE levels (p < 0.001 and p < 0.025, respectively). AGE stimulation increased the expression of RAGE (p = 0.002), ICAM-1 (p = 0.010) and VCAM-1 (p = 0.002) in endothelial cells; TIMP-1 (p = 0.003) and MCP-1 (p = 0.005) in fibroblasts. AGE stimulation increased protein levels of IL-6 (p < 0.001) in endothelial cells; VEGF (p = 0.025) and IL-8 (p < 0.001) in fibroblasts; IL-1b (p < 0.001) and VEGF (p = 0.007) in epithelial cells.

**Conclusion:**

Activation of RAGE pathway may contribute to asthma pathogenesis by increasing the expression of several asthma-related genes. These findings suggest that suppression of RAGE signaling may be an alternative candidate for treating asthma.

## 1. Introduction

Asthma is the most common and highly heterogeneous chronic inflammatory lung disease seen in modern industrial societies. It is estimated to affect more than 300 million people worldwide and cause about 250,000 deaths annually.

Examination of lung tissue of asthmatic patients revealed that the epithelial cell damage, increased basement membrane thickness, increase in airway smooth muscle cells, goblet cell metaplasia and increased mucus production and persistent inflammation are the most prominent pathological features of asthma.

Studies have revealed that heterogeneity is caused by different underlying mechanisms and has led to the concept of endotypes and endotypes specific treatment. Hence, elucidation of different pathophysiological mechanisms at the cellular and molecular level is important for the treatment of various endotypes of the disease.

Although asthma was previously thought to be a Th2 type inflammatory disease, recent studies have shown that new T cell subsets and various cytokines/chemokines secreted from these cells, as well as mast cells, basophils, eosinophils, and recently identified native lymphoid type 2 cells, which are part of the innate immune response, and also structural cells such as epithelial cells, smooth muscle cells and fibroblasts have revealed that they play an important role in the pathology of asthma [1].

As the first barrier to the outside environment, airway epithelial cells serve a central role in the initiation and development of airway inflammation. Chemokines secreted from structural cells are the important molecules for the recruitment and migration of inflammatory cells into the airway submucosa which is the basis of airway hyperresponsiveness (AHR) and airway remodeling [1]. The respiratory system has a large surface area in contact with the environment. Inhalation of cigarette smoke, airborne pollutants (ozone, nitrogen dioxide, sulfur dioxide), or airborne particulate matter can trigger asthma symptoms [2]. It has been reported that various environmental factors such as allergens, viruses, cigarette smoke, and ozone activate innate immunity through pattern recognition receptors (PRR), causing the pathological changes observed in asthma. RAGE (receptor of advanced glycation end products) is one of these pattern recognition receptors and plays an important role in the pathogenesis of asthma [3–6]. Advanced glycated end products which is the one of the well-known RAGE ligand are formed by nonenzymatic glycation or oxidation of amino groups of proteins, lipids and nucleic acids under oxidant stress or hyperglycemia [7–10]. Although connective tissue matrix proteins such as collagen or basement membrane proteins are the main targets, they can also affect proteins such as myelin, complement 3, tubulin, plasminogen activator and fibrinogen [11–14].

Genome-wide association studies have shown that genetic variants in the RAGE gene are associated with the narrowing of the airways. The relationship between RAGE and RAGE ligands in asthma has been investigated before. Some polymorphisms in the exonic and the regulatory regions of the RAGE gene cause changes in gene expression and protein levels and are associated with asthma [15–17]. The level of soluble RAGE, which shows a neutralizing effect for RAGE ligands, was shown to decrease in the mouse model of neutrophilic asthma and was found in lower levels in bronchoalveolar lavage samples of patients with neutrophilic asthma following allergen challenge and patients with acute bronchiolitis compared to controls [5,18,19]. In addition, RAGE ligand HMGB1 was increased in the sputum of patients with asthma and its levels are correlated with disease severity, eosinophil count, and total inflammatory cell count [20]. Recent studies have shown that the RAGE signal is overactive in asthmatics and is associated with disease severity. Although the role of RAGE in the pathogenesis of asthma is known, there is not enough information about the relation between RAGE and airway inflammation triggered by structural cells of the airway and also the role of RAGE in the development of asthma and childhood asthma.

In this study, we investigated the association of two of the described polymorphisms, −374 T/A and Gly82Ser which significantly influence the function of RAGE, and plasma levels of RAGE with childhood asthma. Besides the association study, we also conducted a functional study to reveal its relationship with inflammation and remodeling important in the development of asthma. For this purpose, we investigated the cellular responses that occur in airway structural cells such as epithelium, endothelial, and fibroblasts as a consequence of the RAGE signaling pathway activation.

## 2. Materials and methods

### 2.1. Study population

Asthmatic patients aged between 6 and 18 who were followed up with the diagnosis of asthma in Hacettepe University Pediatric Allergy and Asthma Unit between 2002 and 2014 and control group who presented to the outpatient department of the same hospital for reasons such as minor trauma or their regular follow-up and had never had a history of any allergic disease and or atopy were enrolled to the study in a 3 to 1 ratio. In asthma group inclusion criteria was having doctor diagnosed asthma and exclusion criteria was having systemic disease and chronic inflammatory disorders other than asthma, allergic rhinitis and atopic dermatitis, having neuropsychological disorders and also not accepting the signing of informed consent form. The diagnosis of asthma was made based on history and lung function tests according to the Global Initiative for Asthma (GINA) [21]. DNA and serum samples were obtained from the study subjects with the permission of the Hacettepe University Ethics Committee (Decision No: HEK04/111-17, Annex-2 and Decision No: FUND 02/24-1, Annex-3).

The characteristics of the study population are summarized in Table 1.

### 2.2. Genotyping

Gly82Ser (rs2070600, NC_000006.12:g.32183666C>T) and −374 T/A (rs1800624, NC_000006.12:g.32184610A>T) polymorphisms in the RAGE gene which are affecting protein levels and expression levels of RAGE according to the literature [7,8] were genotyped in 362 asthmatic children and 134 healthy controls by PCR-RFLP analysis. The relevant gene regions were amplified by using following primers; F: 5′-CTTGGAAGGTCCTGTCTCC-3′ and R: 5′-GCCAAGGCTGGGGTTGAAG-3′ for Gly82Ser polymorphism and F: 5′-CCTGGGTTTAGTTGAGATTTTTT-3′ and R:5′-GAAGGCACTTCCTCGGGTTCT-3′ for −374 T/A polymorphisms. Condition of amplifications were follows: 35 cycles of 94 °C for 30 s, 60 °C for 30 s and 72 °C for 30 s. PCR products were digested with Alu I at 37 °C and Tsp509 I (Tas I) at 65 °C for overnight by using 5 unit of enzymes for Gly82Ser and −374 T/A polymorphisms, respectively [8, 22]. The products of enzyme digestions were separated on 3% agarose gel visualized by a UV transilluminator.

### 2.3. Measurement of plasma sRAGE levels

Amounts of RAGE protein in plasma were determined according to the instructions of manufacturers in randomly selected asthmatic and control samples using a commercially available Human RAGE Quantikine ELISA Kit (Quantikine, R&D, Minneapolis, USA) [10].

### 2.4. Cell cultures

BEAS-2B (ATCC CRL-9609), an immortalized human lung epithelial cell line, CCD-16Lu (ATCC CCL-204) human lung fibroblast cell line and HULEC-5a (ATCC CRL-3244) microvascular endothelial cell that was isolated from the lungs were used as a sources of lung structural cells. BEAS 2B cells were grown in DMEM and fibroblasts were grown in DMEM+GlutaMax (Gibco, Thermo Fisher Scientific, UK) supplemented with 10% FBS+1% Pen/Str at 5% CO_2_ at 37 °C. Cells were trypsinized when the cells reach 70%–80% confluent and seeded in 24-well plates at 1 × 10^5^ cells/well. Endothelial cells were grown using the VascuLife VEGF Endothelial Medium Complete Kit (Lifeline Cell Technology, Maryland, USA). Cells were trypsinized and seeded at 0.5 × 10^5^ cells/well. The next day cells were stimulated with different doses of AGE-BSA (50 μg/mL, 100 μg/mL, 200 μg/mL) which are chosen according to the literature [23–26] (BioVision Inc. Milpitas, CA, USA) or control BSA was used as an unmodified protein source (BioVision Inc. Milpitas) for 24 h, and then the supernatants were collected and stored at −80 °C until used in ELISA experiments. RNA was isolated from cells and stored at −80 °C until used for cDNA synthesis.

### 2.5. RNA extraction and determination of gene expressions by real-time PCR

RNA isolation was performed using the GeneJet RNA Purification Kit (Thermo Fisher Scientific, Vilnius, Lithuania) according to the company’s instructions. The purity and concentrations of the obtained RNA samples were determined using NanoDrop. Two hundred and fifty nanograms of RNA samples were translated into complementary DNA using the RevertAid First Strand cDNA Synthesis Kit (Thermo Fisher Scientific). The expression levels of the genes were determined by the real-time PCR using the Luminaris Color HiGreen qPCR Master Mix, low ROX kit (Thermo Fisher Scientific), and 7500 Fast Real-Time PCR system (Applied Biosystems, Waltham, MA, USA).

The changes in the expressions of RAGE, ICAM, VCAM, VEGF genes in endothelial cells, and RAGE, TNF-a, MCP-1, MMP-9, TIMP-1, VEGF, and IL-8 genes in fibroblasts, and RAGE, TNF-a, IL-1b, IL-6, IL-8, and VEGF genes in epithelial cells were determined by real-time PCR and calculated by the 2-ΔΔCT method. Elongation factor 1-alpha (EF1a) was used as a housekeeping gene for normalization of results in real-time PCR experiments. Relevant gene expressions in unstimulated cells were accepted as 1, and changes resulting from stimulation were given as fold increase or decrease. Whether the changes between the groups were statistically significant was determined by statistical comparison of the DCT values of 4 separate experimental sets belonging to each group. List of the primers used in Real-Time PCR was given in Table 2.

### 2.6. Measurement of RAGE, cytokine/chemokine levels in cell culture supernatants by ELISA

Protein levels of IL-6, IL-8, TNF-a, ICAM, VCAM, VEGF in endothelial cells, and IL-6, IL-8, TNF-a, MCP-1, MMP-9, TIMP-1, VEGF in fibroblasts, and TNF-a, IL-1b, IL-6, IL-8,, MMP-9, TIMP-1, VCAM, and VEGF in epithelial cells were determined in the supernatant of control and AGE-BSA stimulated cells using commercially available colorimetric ELISA kits (TIMP-1 kit purchased from BT LAB, Shanghai, China, the others purchased from Invitrogen, Carlsbad, CA, USA) according to the manufacturer’s instructions.

### 2.7. Statistical methods

Statistical analyses were done using SPSS 22 for Windows program. The allele frequency of the Gly82Ser and −374 T/A polymorphisms in the RAGE gene was compared in asthmatic and normal cohorts. In addition, the relationship of these polymorphisms with important asthma phenotypes such as eosinophils, IgE, and FEV1 was statistically evaluated. Comparison of numerical variables such as age, eosinophil count, IgE levels, % FEV1 values, serum protein measurements was made according to the distribution of data and the ones normally distributed were analyzed by parametric tests and the others by nonparametric tests, respectively. Categorical variables were evaluated using the chi-square test or Fisher’s exact test. The qPCR results were compared between the controls and groups using the 2-ΔΔCT method. The results are given as fold change. Whether the changes in gene expressions were statistically significant or not was determined by comparing the ΔCT values of 4 samples in each group using parametric tests. In terms of both expression analysis and protein levels in cell culture supernatants comparison between/within groups made by using one-way ANOVA method. Statistically significant parameters in the ANOVA test were compared with the independent sample t-test, and the difference between the groups was determined. In all analyses, p < 0.05 was considered statistically significant.

## 3. Results

### 3.1. RAGE genotyping and plasma levels

**Figure 1:**
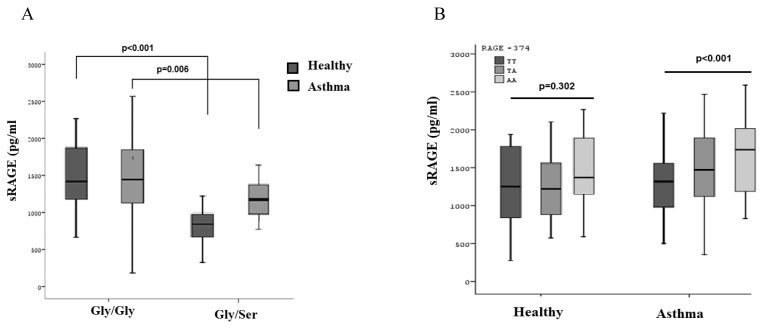
Plasma sRAGE levels of asthmatics and healthy controls according to Gly82Ser (A) and −37T/A (B) genotyping.

Plasma levels of sRAGE were significantly lower in both healthy and asthmatic children carrying the serine allele compared to those carrying the glycine allele (healthy children: 1450 pg/mL in glycine allele carriers vs. 811 pg/mL in serine carriers; asthmatic children: 1488 pg/mL in glycine allele carriers vs. 978 pg/mL in serine carriers) (p < 0.001) (Figure 1A). Similarly lower plasma sRAGE levels were observed in T allele carriers of −374 polymorphism compared to carriers of the A allele in the T dominant model in asthmatics (1299 pg/mL in TT and TA carriers and 1722 pg/mL in AA carriers) (p = 0.025) (Figure 1B). Frequencies of the Gly and Ser alleles in healthy controls and asthmatic children were same (Gly = 0.96 and Ser = 0.04). Frequency of −374 T allele was 0.58 (0.42 for A allele) in healthy controls while 0.61 (0.39 for A allele) in asthmatic children. However, there was no difference in the frequency of genotypes or plasma levels of sRAGE between asthmatic and healthy children. Other allergic phenotypes were not associated with the studied polymorphisms.

### 3.2. Real-time PCR results

#### Endothelial cells

**Figure 2:**
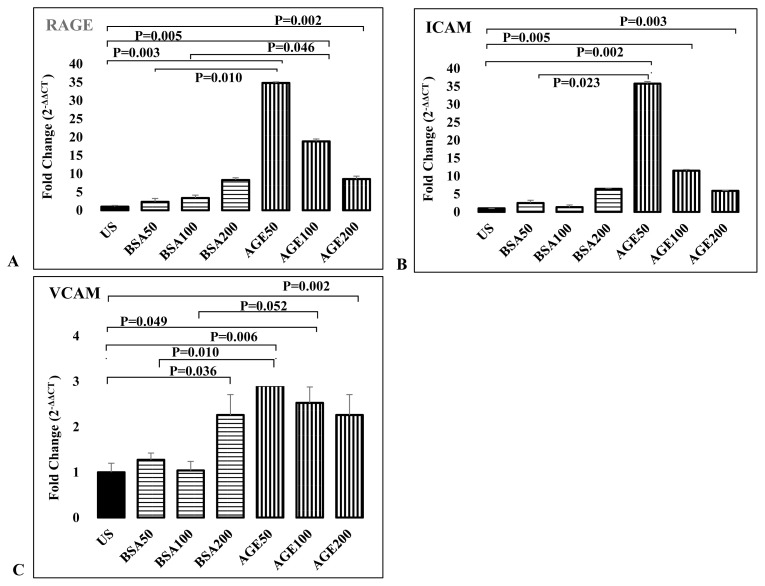
Effects of activation of RAGE signaling by AGE-BSA stimulation on expressions of inflammation related genes from endothelial cells. Comparison of RAGE (A), ICAM (B), and VEGF (C) genes expressions in endothelial cells stimulated with different concentrations (50 μg/mL, 100 μg/mL, 200 μg/mL) of control BSA and AGE-BSA.

Stimulation with AGE-BSA significantly increased RAGE expression compared to both unstimulated and control BSA-stimulated samples. In addition, this change decreased with increasing doses of RAGE (50 μg/mL: 35-fold (p = 0.003), 100 μg/mL: 19-fold (p = 0.005), 200 μg/mL: 8.5-fold (p = 0.002)). Stimulation with both 50 μg/mL and 100 μg/mL AGE-BSA significantly increased the expression of the RAGE gene compared to the stimulations with control BSA at the same doses (p = 0.010 and p = 0.046, respectively) (Figure 2A). Stimulation with AGE-BSA caused a dose-related increase in ICAM gene expression compared to unstimulated samples (50 μg/mL: 36-fold (p = 0.002), 100 μg/mL: 11.5-fold (p = 0.003), 200 μg/mL: 6-fold (p = 0.005)) (Figure 2B). Compared to stimulation with control BSA at the same dose, only 50 μg/mL AGE-BSA significantly increased ICAM gene expression compared to control BSA (p = 0.023). All three doses of AGE-BSA stimulation significantly increased VCAM expression compared to unstimulated condition (50 μg/mL, p = 0.010; 100 μg/mL, p = 0.049; 200 μg/mL, p = 0.002). When compared to BSA controls, significance was observed only at 200 μg/mL control BSA (Figure 2C).

#### Fibroblast cells

**Figure 3:**
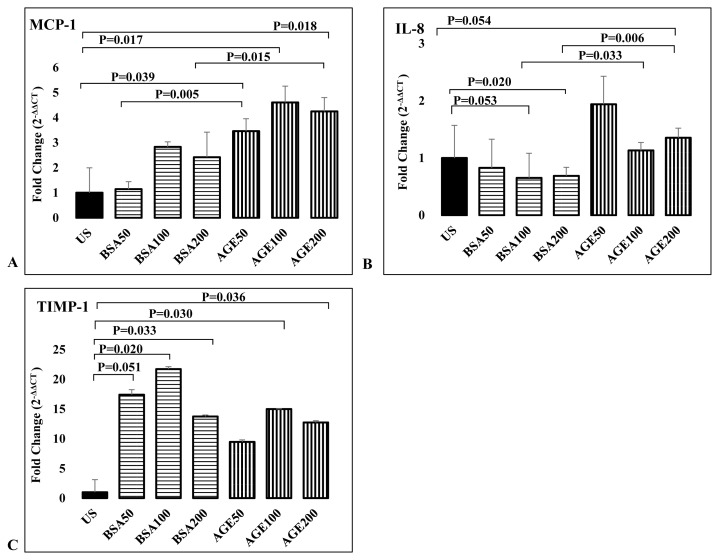
Effects of activation of RAGE signaling by AGE-BSA stimulation on expressions of inflammation related genes from fibroblasts. Comparison of expressions of MCP-1 (A), IL-8 (B), and TIMP-1 (C) genes in fibroblasts stimulated with different concentrations (50 μg/mL, 100 μg/mL, 200 μg/mL) of control BSA and AGE-BSA.

MCP-1 gene expression increased significantly in all three doses compared to unstimulated samples (50 μg/mL: 3.5-fold (p = 0.039), 100 μg/mL: 4.6-fold (p = 0.017), 200 μg/mL: 4.25-fold (p = 0.018)) (Figure 3A). Compared to control BSA, stimulation with 50 μg/mL and 200 μg/mL AGE-BSA significantly increased MCP-1 gene expression compared to controls (p = 0.005 and p = 0.015, respectively). While stimulation with 50 μg/mL AGE-BSA increased IL-8 gene expression 1.9 times compared to unstimulated samples, this increase failed to reach significance. However, when stimulation with 100 and 200 μg/mL AGE-BSA was compared with stimulation with the same dose control BSA, a significant difference was found between the groups (p = 0.033 and p = 0.006, respectively) (Figure 3B). TIMP-1 gene expression increased significantly with both stimuli (Figure 3C). Similarly, MMP-9 gene expression decreased but failed to reach significance.

#### Epithelial cells

Stimulation with different doses of control BSA caused a significant reduction in RAGE TNF-a, IL-1b, IL-6, and IL-8 gene expressions (data not shown).

### 3.3. ELISA results

**Figure 4:**
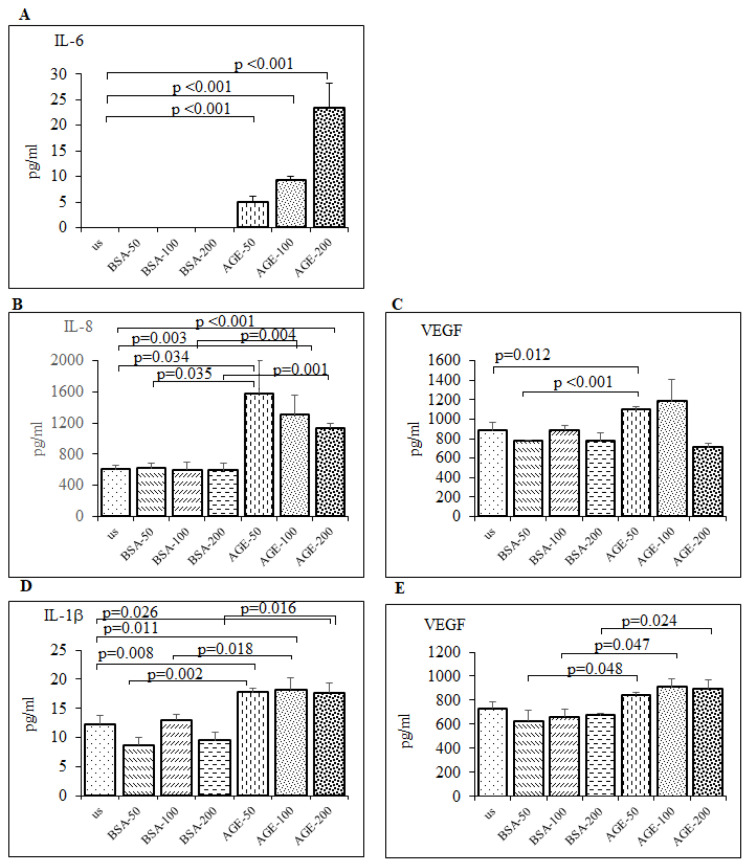
Effects of AGE-BSA stimulation on release of cytokines/chemokines and growth factors from structural cells. (A) IL-6 protein levels in endothelial cells, (B) IL-8 and (C) VEGF protein levels in fibroblasts, (D) IL-1b and (E) VEGF protein levels in bronchial epithelial cells after stimulation with different concentrations (50 μg/mL, 100 μg/mL, 200 μg/mL) of control BSA and AGE-BSA. (Only the statistically significant changes in protein levels were given in the figure.)

IL-6, IL-8, TNF-a, VCAM, and VEGF protein levels were investigated in the supernatants from endothelial cells by the ELISA method. TNF-a and VCAM proteins were not within the measurable range. Stimulation with control BSA and AGE-BSA did not cause a significant increase in IL-8 and VEGF protein levels, but IL-6 levels increased significantly compared to unstimulated and control BSA-stimulated samples (p < 0.001) (Figure 4A).

In fibroblasts, stimulation with control BSA and AGE-BSA did not change IL-6, MCP-1, and TIMP-1 protein levels but increased IL-8 and VEGF levels significantly (Figures 4B and 4C, respectively).

In epithelial cells, stimulation with control BSA and AGE-BSA did not cause a significant increase in IL-8, MCP-1, MMP-9, and TIMP-1 protein levels, but resulted in a significant increase in IL-1b and VEGF levels (Figures 4D and 4E, respectively). However, the increase in VEGF protein level as a result of stimulation with AGE-BSA was not statistically significant compared to unstimulated samples, but only with control BSA.

## 4. Discussion

RAGE, the advanced glycation end products receptor, is one of the few pattern recognition receptors that can recognize both PAMPs and DAMPs. RAGE contributes to both inflammatory responses and tissue remodeling [27].

Although the role of RAGE in the pathogenesis of asthma is known, there is not enough information about the relation between RAGE and airway inflammation triggered by structural cells of the airway.

In this study first we performed molecular study to reveal whether the RAGE polymorphisms is associated with asthma. For this purpose we chose two variants most prominent ones which can affect protein levels or functions of RAGE molecule according to the literature. Our results showed that the Gly82Ser and −374 T/A polymorphisms in RAGE gene were associated with lower plasma soluble RAGE (sRAGE) levels (p < 0.001 and p < 0.025, respectively). Since the sRAGE molecule acts as a neutralizing antibody for RAGE ligands we thought that in the carriers of these variant inflammatory response may change. Because of the difficulties of the get bronchoscopy samples from patients who carry different alleles of each variants we used cell lines as a source of structural cells for our experiments to show inflammatory responses as a result of activation of RAGE pathway in the second parts of the study.

We investigated the cellular response of airway structural cells as a result of activation of the RAGE signaling pathway triggered by the AGE ligand. We found an increase in the expressions of ICAM and VCAM genes in endothelial cells related to RAGE. The stimulation of endothelial cells with AGE increased the expression of the RAGE and ICAM-1 genes in a dose-dependent manner. The expression of VCAM-1, also increased, although not as prominently as in the RAGE and ICAM genes. In addition, IL-6 protein levels increased significantly after stimulation with AGE in a dose-dependent manner in this cell.

ICAM-1 is a cell surface glycoprotein typically expressed on endothelial cells and immune system cells. Activated leukocytes bind to endothelial cells via ICAM-1/LFA-1 and then migrate to tissues [28,29]. ICAM-1 has also been identified as a mediator for the cellular entry of human rhinovirus. In primary endothelial cell cultures derived from a human leg vein, binding of RAGE with AGEs resulted in an increase of expression of VCAM-1, ICAM-1, and E-selectin and an increase in the amount of these molecules on the cell surface, while at the same time increasing the adhesion of polymorphonuclear leukocytes to stimulated endothelial cells [30]. In another study, it was shown that the binding of AGEs to RAGE in human endothelial cells activates the Rho signaling pathway, causing cytoskeletal rearrangements, and these rearrangements, resulting in increased vascular permeability, allow the transmigration of leukocytes [31].

Another molecule which increased after stimulation by AGE in endothelial cells is IL-6 is a cytokine that acts as an important regulator of effector CD4 T cell differentiation. It inhibits Th1 differentiation by promoting IL-4 production during Th2 differentiation and, together with TGFβ, promotes Th17 cell differentiation [32]. Because of these immune-regulatory roles, IL-6 is thought to be an important contributor to the pathogenesis of asthma and other pulmonary diseases with epithelial damage.

Since the ICAM and VCAM triggers inflammation by causing an increase in the transmigration of leukocytes into the tissue [28,29,33,34] and IL-6 is a regulator of effector CD4 T cell differentiation according to the literature we may speculate that activation of RAGE signaling pathway in endothelial cells via AGE stimulation leads to increased migration of inflammatory cells into the tissue through ICAM and VCAM and may also contribute to the increase and persistence of inflammation by promoting the differentiation of T lymphocytes via IL-6.

Fibroblasts, another structural cell group important in remodeling and asthma, are activated during tissue repair processes and transform into myofibroblasts, which are the main source of extracellular matrix (ECM) proteins [35,36]. ECM proteins play important roles in regulating many functions, including cell growth and migration, cytoskeletal organization, and tissue development [37]. Abnormal deposition of ECM has been observed in the submucosal areas of the large and small airways of asthmatic patients [38–40]. Disruption of the ECM occurs during tissue remodeling. Matrix metalloproteinases (MMPs) are an important group of enzymes that degrade ECM proteins. The degradation of ECM by MMPs is controlled by TIMPs, known as tissue inhibitors of MMPs, to prevent tissue damage that can be caused by excessive degradation.

In fibroblasts, stimulation with the RAGE ligand AGE caused an increase in angiogenic factors such as VEFG, MCP-1, IL-8, as well as a decrease in MMP-9 and an increase in TIMP-1 (low MMP-9/TIMP-1 ratio). In epithelial cells, another structural cell of the airway, the protein levels of IL-1β and VEGF increased with AGE stimulation.

MMP-9 can be synthesized by bronchial epithelial cells, endothelial cells, fibroblasts, alveolar macrophages, mast cells, dendritic cells, neutrophils, and eosinophils [41]. It is well known that MMP-9 induces an angiogenic change in carcinogenesis [42,43]. Lee et al. showed that the VEGF signaling pathway was effective in regulating MMP-9 gene expression using a mouse model of asthma, and VEGF receptor inhibition caused a decrease in MMP-9 expression [44]. The imbalance between MMP-9 and TIMP-1 is recognized as an important theory to explain the development of airway remodeling in asthma. TIMP-1 antagonizes MMP-9 activity, and the finding of lower MMP-9/TIMP-1 ratios in the sputum of smokers with asthma suggests that excessive TIMP-1 production occurs as a defense against MMP-9 production in these patients [45].

In our study, stimulation of fibroblast cells with AGE caused a decrease in the expression of TNF-a and MMP-9 and an increase in the expression of TIMP-1 and MCP-1 genes, but the decrease in TNF-a and MMP-9 was not statistically significant. In addition, stimulation with AGE also caused a significant increase in IL-8 and VEGF protein levels.

Monocyte chemoattractant protein-1 (MCP-1) is the most important chemokine that regulates the migration and infiltration of monocytes/macrophages. Monocyte chemoattractant protein-1 (MCP-1) has also been recognized as an angiogenic chemokine [46,47]. Hong et al. showed in their research that MCP-1 increases VEGF-A gene expression and VEGF-A causes an increase in angiogenesis [48].

IL-8, also known as a neutrophil chemotactic factor, induces chemotaxis in neutrophils and other granulocytes, causing these cells to migrate toward the infection site. IL-8 is also known to be a potent promoter of angiogenesis.

When the findings we obtained as a result of our study are evaluated together with the data in the literature, it supports that the activation of the RAGE signaling pathway triggers the development of angiogenesis and remodeling which are the important features of in asthma.

The proinflammatory cytokine interleukin (IL)-1β produced by the airway epithelium is thought to play an important role in the pathogenesis of asthma [49]. It has been shown that IL-1β levels increase in the serum and sputum of patients with asthma and this increase is associated with the severity of airway dysfunction [50,51]. Osei et al. investigated the effect of IL-1 cytokines released from the airway epithelium on fibroblast-induced inflammation, extracellular matrix, and collagen remodeling by using primary airway fibroblasts and they showed that IL-1α/β induces proinflammatory responses in fibroblasts and suppresses ECM production and the cell’s ability to repair and remodel fibrillar collagen I [52].

When epithelial cells were stimulated with AGE, no significant increase in gene expression was observed compared to the nonstimulated cells. Only the expression level of the IL-1β decreased, while the protein levels of IL-1β increased significantly in cell culture supernatants. We think that the reason why the increase we observed in IL-1β protein level was not observed in the expression level in our study was since both RNA and supernatant samples were obtained after 24 hours of stimulation, and the increase in IL-1β gene expression occurred in the early hours of the stimulation and then decreased.

In previous in vitro studies, AGE caused an increase in proteins such as PPAR-g, IL-1β, AP-1, adhesion molecules such as VCAM-I, ICAM, and growth factors such as VEGF and IGF-I, angiogenic and proinflammatory cytokines [53–56].

Briefly, in our study, stimulation with the RAGE ligand AGE caused an increase in angiogenic factors such as VEFG, MCP-1, IL-8 in fibroblasts, as well as a decrease in MMP-9 and an increase in TIMP-1 (low MMP-9/TIMP-1 ratio). The increase in both the level of IL-1β protein released from the endothelium and the expression of VEGF makes us think that the activation of the RAGE signaling pathway contributes to the development of remodeling and angiogenesis. In addition, we can say that the activation of the RAGE signaling pathway contributes to the increase in the transmigration of leukocytes to the inflammation area by causing an increase in ICAM gene expression in endothelial cells, and to the formation of Th2 type inflammation by stimulating Th2 cell differentiation with the increase of IL-6. Besides, the fact that RAGE increases ICAM gene expression, which is an entry point for respiratory viruses into the cell, made us think that RAGE may also be effective in virus-induced asthma attacks in asthma. We believe that further studies to be designed on this subject will contribute to the elucidation of the role of RAGE in the pathogenesis of asthma and to obtain new evidence to support its use as a treatment candidate.

The limitations of our study are that coculture models were not used in our study. In living tissue, these cells coexist and interact with each other through cell-cell contact or via the secreted molecules. It will be much better to use a three-dimensional cell culture model to replicate our results and confirm the findings. In this way, it will be possible to better understand the pathogenesis of the disease and the role of RAGE in this pathogenesis.

## 5. Conclusion

The data we obtained in our study supports that polymorphisms in the RAGE gene are associated with lower plasma RAGE levels and activation of the RAGE signaling pathway is involved in various asthma-related pathophysiological mechanisms such as migration of inflammatory cells into the tissue, Th2 cell differentiation, and airway remodeling and angiogenesis. In the light of all these data, we believe that molecules such as RAGE antagonists or neutralizers, which suppress the RAGE activation pathway, can be an effective treatment alternative because they can simultaneously block several different pathways in the pathogenesis of asthma.

## Figures and Tables

**Table 1:** The demographics and clinical characteristics of the study population.

	Asthma (n = 362)	Controls (n = 134)
Age (year)	10.6 (8.1–13.6)	10.7 (8.1–13.1)
Sex %(F /M)	36/64	52/48
Atopy %	63	16
FEV1	94 (84–103)	
FEV/FVC	88 (82–92)	
Total IgE	176 (65–461)	
Eosinophil number	285 (170–470)	
Eosinophil %	3.7 (2.2–6.0)	

**Table 2:** List of real-time PCR primers.

IL6	F: 5′-GCAGATGAGTACAAAAGTCCTGA-3′ R: 5′-TTCTGTGCCTGCAGCTTC-3′
IL8	F: 5′-CTGCGCCAACACAGAAATTAT-3′ R: 5′-AAACTTCTCCACAACCCTCTG-3′
MMP9	F: 5′-ACATCGTCATCCAGTTTGGTG-3′ R: 5′-CGTCGAAATGGGCGTCT-3′
TIMP1	F: 5′-TGTTTATCCATCCCCTGCAAA-3′ R: 5′-CAAGGTGACGGGACTGGAA-3′
IL1-b	F: 5′-TTGAAGCTGATGGCCCTAAA-3′ R: 5′-TGAACCCCTTGCTGTAGTG-3′
TNF-a	F: 5′-CCAGGGACCTCTCTCTAATCA-3′ R: 5′-TCAGCTTGAGGGTTTGCTAC-3′
MCP-1	F: 5′-AGCAAGTGTCCCAAAGAAGC-3′ R: 5′-CATGGAATCCTGAACCCACT-3′
RAGE	F: 5′-CAACGGCTCCCTCTTCCTT-3′ R: 5′-TTGGTCTCCTTTCCATTCCTGT-3′
VEGFA	F: 5′-GCAGAATCATCACGAAGTGGTG-3′ R: 5′-TCTCGATTGGATGGCAGTAGCT-3′
VCAM-1	F:5′-TTCCCTAGAGATCCAGAAATCGAG-3′ R:5′-CTTGCAGCTTACAGTGACAGAGC-3′
ICAM	F: 5′-AACCAGAGCCAGGAGACACTG-3′ R: 5′-GCGCCGGAAAGCTGTAGATG-3′
